# Mid- to long-term outcomes of covered balloon-expandable stent implantation for the management of vascular injuries in patients undergoing transfemoral transcatheter aortic valve implantation: the BE-SAFE Registry for the RECOVER (REsults after percutaneous interventions with COVERed stents) Investigators

**DOI:** 10.1007/s00392-025-02651-2

**Published:** 2025-05-15

**Authors:** Hector A. Alvarez-Covarrubias, Martin Jurisic, Finn Syryca, Charlotte Duesmann, Costanza Pellegrini, Tobias Rheude, N. Patrick Mayr, Niklas Altaner, Moritz Kühlein, Tobias Lenz, Edna Blum, Yousuke Taniguchi, Gjin Ndrepepa, Christian Thilo, Heribert Schunkert, Adnan Kastrati, Sebastian Kufner, Salvatore Cassese, Michael Joner, Erion Xhepa

**Affiliations:** 1 Department of Cardiology, TUM University Hospital German Heart Center, Munich, Germany; 2 Hospital de Cardiología, Centro Médico Nacional Siglo XXI, IMSS, Mexico City, México; 3 Institut für Anästhesiologie, Deutsches Herzzentrum München, Technische Universität München, Munich, Germany; 4 Department of Cardiology, RoMed Klinikum Rosenheim, Rosenheim, Germany; 5 DZHK (German Center for Cardiovascular Research), Partner Site Munich Heart Alliance, Munich, Germany

**Keywords:** Covered balloon-expandable stent, Vascular complication, Transcatheter aortic valve implantation, Transfemoral access

## Abstract

**Background and aims:**

Vascular complications occur in a non-negligible proportion of transfemoral transcatheter aortic valve implantation (Tf-TAVI) procedures. There is only limited evidence regarding the efficacy and safety of covered balloon-expandable (CBE) stents in the management of Tf-TAVI related vascular complications. We aimed to investigate the efficacy and safety of CBE stent implantation to treat access-related vascular complications in patients undergoing Tf-TAVI.

**Methods:**

The present retrospective analysis included patients undergoing CBE stent implantation following Tf-TAVI from April 2012 to January 2023 at our centre. The primary endpoint was technical success defined as successful device delivery and implantation at the intended location with angiographic confirmation of vessel patency and absence of residual bleeding. Clinical outcomes and color Doppler ultrasonography findings were evaluated at discharge, 30 days and longest available follow-up.

**Results:**

Among 3331 Tf-TAVI procedures, 93 patients (2.8%) required covered stent implantation for the treatment of access related vascular complications. Technical success was achieved in 92 patients (98.9%). BeGraft and Atrium Advanta V12 CBE stents were implanted in 76 (81.7%) and 17 (18.3%) patients, respectively. Median clinical follow-up was 455 [304; 798] days, with both in-hospital and 30-days mortality equaling 4.3% and 1-year mortality 15.1%. Pre-discharge Doppler ultrasonography was performed in 98.9% patients, with normal findings in 79.8% and minor findings (such as pseudoaneurysm, non-flow-limiting dissection, arterio-venous fistula) in the remaining patients. Clinical follow-up was available in 96.2% patients; no cases of new-onset claudication, need for repeat surgical or transcatheter vascular interventions were recorded. Follow-up Doppler ultrasonography (54.4% patients) showed persistent patency and no signs of stent failure in all patients.

**Conclusions:**

CBE stent implantation for the treatment of access site related vascular complications after Tf-TAVI is associated with excellent technical success rates, optimal short- to mid-term patency rates as well as satisfactory long-term clinical outcomes.

**Supplementary Information:**

The online version contains supplementary material available at 10.1007/s00392-025-02651-2.

## Introduction

A transfemoral (Tf) approach currently represents the access of choice in patients undergoing transcatheter aortic valve implantation (TAVI) for severe aortic valve stenosis (AVS) [1, 2]. Although continuous device iterations, increasing operator experience and expansion of TAVI indications towards lower risk populations have led to a drastic reduction in the rate of access-related major vascular complications, the latter still occur in a non-negligible proportion of procedures and have a negative impact on patient outcomes [3–5]. 

Prompt recognition as well as management of large-bore access-related vascular complications is of paramount importance, with available treatment options ranging from prolonged manual compression, endovascular balloon inflation, covered stent implantation or surgical vascular repair [6]. Since the vast majority of vascular complications are recognized at the end of the procedure with a contralateral arterial access already available, an endovascular approach represents an attractive treatment strategy, which might even have potential advantages compared to conventional surgical repair [7]; indeed, considering the advanced age and comorbidities of patients undergoing TAVI procedures, the latter option portends a non-negligible morbidity and mortality risk.

If implantation of covered stents is required, both covered self-expanding (CSE) and covered balloon-expandable (CBE) stent platforms are available with the choice being usually dictated by operator’s expertise and preferences [8]. Currently, the evidence supporting use of covered stent platforms in the management of large-bore access-related vascular complications in the setting of TAVI is limited to small retrospective registries, mostly reporting short- to mid-term outcomes following CSE stents [9–12], while no direct comparative studies evaluating efficacy and safety of CSE and CBE stent platforms have been performed so far. Against this background, we aimed to investigate the efficacy and safety of CBE stent implantation for the treatment of access-related vascular complications in patients undergoing Tf-TAVI in a large, all-comers retrospective patient cohort.

## Methods

### Study population

This is a retrospective, observational analysis including all consecutive patients undergoing implantation of covered stents for the treatment of access site related vascular complications in the setting of Tf-TAVI between April 2012 and January 2023 at the Department of Cardiology of the German Heart Centre Munich, Germany.

Patient clinical and procedural information was extracted from a dedicated, prospective institutional database. During the study period, 3331 Tf-TAVI procedures were performed at our centre using commercially available transcatheter heart valve (THV) systems. Indication for TAVI was discussed in a multidisciplinary heart team, after careful consideration of patient age and clinical characteristics as well as findings of transthoracic echocardiography, multi-slice computed tomography (MSCT) and coronary angiography.

The study was performed in accordance with the principles of the Declaration of Helsinki and all patients provided written informed consent for the procedure. Ethical approval was obtained from the Ethics Committee of the Technical University of Munich under the OBSERVTAVI Registry (#525/17).

### Computed tomography analysis

MSCT was analyzed by two cardiologists with extensive experience in the evaluation of MSCT. Based on MSCT reconstruction, qualitative and quantitative measures of valve size and peripheral vessel size as well as presence and distribution of calcification were performed by multi-planar reconstruction (MPR) using the 3-Mensio software (Pie Medical Imaging, Maastricht, The Netherlands). Vessel calcification was graded based on the degree of its circumferential extension for each ilio-femoral segment. Vessel angulation was measured at the most acute angle in the abdominal aorta and the iliac artery segments; tangent angle function was applied between two neighbouring vascular segments including the acute angle. Lumen vascular diameter was measured for every aorto-iliac segment and plaque characteristics were also evaluated.

### Procedural technique

**Fig. 1:**
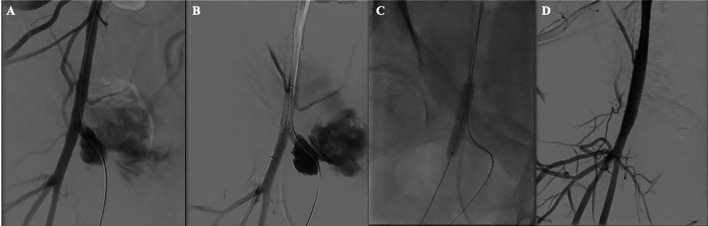
**A** Bleeding complication at the main access vessel following closure device failure; **B** Balloon-expandable covered stent (Bentley 8 × 37 mm) positioning at the bleeding level; **C** Covered stent implantation; **D** Final angiographic result

Vessel access, obtained through a standard percutaneous Seldinger technique in all patients, was followed by the placement of one or two suture-based closure systems (Perclose ProGlide—Abbott Vascular). Following THV implantation, the sheath was retrieved and the suture-based closure systems were tightened; in case of persistent residual bleeding, plug-based closure systems (6 F or 8 F) were applied at the discretion of the operator. In case of major vascular complication not manageable by other means (uncontrollable bleeding and/or vessel stenosis/occlusion/dissection) (Fig. 1A), a cross-over technique was used to gain access to the superficial femoral artery and advance a 0.035 inch supportive guidewire as well as a 7 F/8 F destination guiding sheath in the superficial/common femoral artery of the main access site, respectively, followed by the implantation of covered stents under fluoroscopic guidance (Fig. 1B and C). The size (length and diameter) of the covered stent was chosen based on both CT and angiographic measurements. Finally, a digital subtraction angiography was performed to confirm vessel patency and absence of residual bleeding and/or vascular complications (Fig. 1D).

### Clinical and Doppler ultrasound follow-up

Patients were monitored for the occurrence of adverse events during the hospital stay and a vascular color Doppler ultrasound was recommended before discharge in all patients to exclude silent vascular injury, such as significant vascular stenosis or residual dissections. Clinical follow-up was performed by office visit, phone contact or structured follow-up letter. Doppler ultrasonographic follow-up was recommended for all patients at 1 year.

### Endpoints

The primary endpoint of the study was represented by technical success, defined as successful delivery and implantation of the covered stent at the intended vascular location with angiographic confirmation of vessel patency and absence of residual bleeding, need for bailout surgery or intra-procedural death. In-hospital mortality, new onset claudication, acute limb ischemia or amputation of the ipsilateral extremity, need for repeat percutaneous or surgical vascular intervention as well as evidence of stent occlusion or fracture were also assessed. Vascular complications were evaluated and reported in accordance with the Valve Academic Research Consortium (VARC)−3 criteria [13]. Doppler ultrasonographic data obtained before discharge or during follow-up were recorded in all patients.

### Statistical analysis

Categorical variables are shown as counts (%). Normality of distribution of continuous data was assessed using the Shapiro–Wilk test. Continuous data are shown as mean ± standard deviation (SD) or median with [25 th–75 th] percentiles depending on the distribution pattern. Statistical analysis was performed using SPSS Statistics Version 29.0.

## Results

### Patient population

**Fig. 2:**
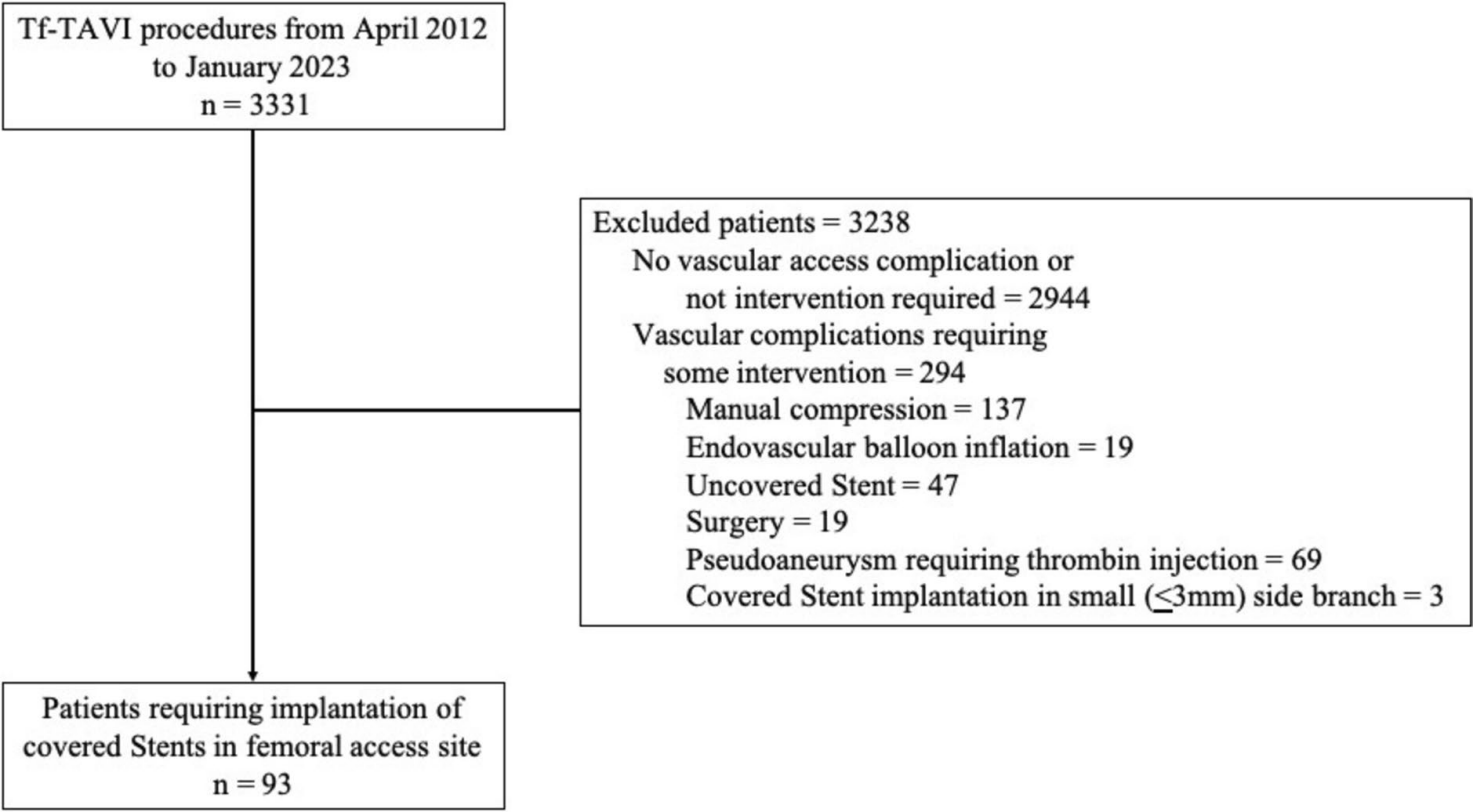
Flowchart of the study population

Between April 2012 and January 2023, 3331 Tf-TAVI procedures were performed at our centre. Minor and/or major vascular complications occurred in 387 patients (11.6%), with covered stent implantation to treat access-site related vascular complications being required in 93 patients (2.8%) (Fig. 2). Patient baseline clinical and echocardiographic characteristics are shown in Table 1. The median [25 th–75 th percentile] age was 81 [77; 85] years and 71% of patients (*n* = 66) had concomitant coronary artery disease. Left ventricular ejection fraction was 56% [45%; 60%] and the majority of patients had a moderate-to-high surgical mortality risk (EuroScore I: 12.64 [8.14; 23.46]; EuroScore II 4.07 [2.49; 7.27]).

**Table 1:** Baseline clinical and echocardiographic characteristics

	*N *= 93
Age, years	81 [77; 85]
Sex, female	59 (63.4)
Body mass index, kg/m^2^	26.9 [24.1; 29.1]
Body surface area, m^2^	1.80 [1.68; 2.0]
New York Heart Association Class ≥ III	57 (61.3)
Arterial hypertension	86 (92.5)
Diabetes mellitus	35 (37.6)
Dyslipidemia	73 (78.5)
Smoking history	9 (9.7)
Atrial fibrillation	51 (54.8)
Chronic obstructive pulmonary disease	10 (10.8)
Peripheral artery disease	25 (26.9)
Previous pacemaker	13 (14)
Coronary artery disease	66 (71)
Previous percutaneous coronary intervention	36 (38.7)
Previous myocardial infarction	9 (9.7)
Previous coronary artery bypass grafting	8 (8.6)
Previous aortic valve replacement	10 (10.7)
Surgical	7 (7.5)
TAVI	3 (3.2)
History of cancer	19 (20.4)
Serum creatinine, mg/dL	1.08 [0.89; 1.43]
Estimated glomerular filtration rate, mL/min	57 [40; 71]
Chronic dialysis	2 (2.2)
Previous stroke/transient ischemic attack	8 (8.6)
Left ventricular ejection fraction, %	56 [45; 60]
Aortic valve area, cm^2^	0.71 [0.55; 0.82]
Mean transvalvular pressure gradient, mmHg	39.8 ± 15.3
Systolic pulmonary artery pressure, mmHg	46.1 ± 14.1
EuroScore I	12.64 [8.14; 23.46]
EuroScore II	4.07 [2.49; 7.27]

Data are shown as counts (%), mean ± SD (standard deviation) or median [25^th^–75 th percentiles]

*TAVI* transcatheter aortic valve implantation

### CT analysis

CT characteristics are reported in Table 2. The mean most acute angle on the main access side was 90 ± 27^°^. Minimal lumen diameter (MLD) at the main access side was 7.2 ± 1.8 mm for the common iliac artery (CIA), 6.1 ± 1.5 mm for the external iliac artery (EIA) and 6.05 ± 1.4 mm for the common femoral artery (CFA). Circumferential vessel calcification (> 180^°^) at the main access side was observed in 38 patients (40.9%) for the CIA, 14 patients (15.1%) for the EIA and 15 patients (16.1%) for the CFA. Aortic valve characteristics are shown in Supplementary Table 1. Aortic valve mean diameter was 23.9 ± 2.4 mm, while severe aortic valve calcification was present in 24.1% of patients.

**Table 2:** Computed tomography analysis of peripheral vessels

	*N* = 93
Most acute iliac vessel angulation, degrees	
Main access	90.7 ± 27.0
Right	89.7 ± 28.5
Left	92.1 ± 27.7
Main access, MLD, mm	5.5 ± 1.4
CIA minimal diameter, mm	7.2 ± 1.8
CIA reference diameter, mm	9.2 ± 1.9
CIA index	0.81 [0.72; 0.94]
EIA minimal diameter, mm	6.1 ± 1.5
EIA reference diameter, mm	7.4 ± 1.6
EIA index	0.87 [0.80; 0.96]
CFA minimal diameter, mm	6.05 ± 1.4
CFA reference diameter, mm	7.4 ± 1.4
CFA index	0.84 [0.72; 0.95]
Circumferential degree of calcification of peripheral vessels	
Main access	
Common iliac artery	
No calcification	4 (4.3)
0 to 180 degrees	51 (54.8)
More than 180 degrees	38 (40.9)
External iliac artery	
No calcification	34 (36.6)
0 to 180 degrees	45 (48.4)
More than 180 degrees	14 (15.1)
Common femoral artery	
No calcification	15 (16.1)
0 to 180 degrees	63 (67.8)
More than 180 degrees	15 (16.1)

Data are shown as counts (%), mean ± SD (standard deviation) or median [25^th^–75 th percentiles]

*CIA* common iliac artery, *CFA* common femoral artery, *EIA* external iliac artery, *MLD* minimum lumen diameter, *mm* millimeters

### Procedural characteristics and in-hospital outcomes

Procedural characteristics are shown in Table 3. The main vascular access was achieved via right or left CFA in 74 (79.6%) and 19 (20.4%) patients, respectively. The minimal lumen diameter was 5.5 ± 1.4 mm, with a sheath to artery ratio of 0.86 [0.71; 1.04]. Sheath size was 14 F in 82 patients (88.2%) and 16 F in 11 patients (11.8%). An upfront percutaneous transluminal angioplasty (PTA) of the iliofemoral vessels to facilitate sheath insertion was required in 10 patients (10.8%). A balloon-expandable THV was implanted in 63.8% of patients while a self-expanding THV was used in the remaining patients. Vascular complications requiring covered stent implantation were located at the main access site in 87 (93.5%) patients and at the contralateral access site in 6 (6.5%) patients. CBE stents were used in all cases, with BeGraft (Bentley InnoMed GmbH, Hechingen, Germany) stents being implanted in 76 (81.7%) and Atrium Advanta V12 (Getinge) stents in 17 (18.3%) patients, respectively. Seventy-eight patients (83.9%) received one covered stent, whereas 2 or more CBE stents were required in 15 (16.1%) patients.

**Table 3:** Procedural characteristics

	*N* = 93
Elective procedure	84 (90.3)
Procedure time, min	90 [70; 111]
Fluoroscopy time, min	20.3 [15.3; 29.3]
Fluoroscopy dose, cGy*cm^2^	4648.1 [2257.5; 7057.6]
Contrast medium, mL	244 [200; 320]
Main access	
Right	74 (79.6)
Left	19 (20.4)
Minimal lumen diameter, mm	5.5 ± 1.4
Sheath to artery ratio	0.86 [0.71; 1.04]
Sheath size (Fr)	
14	82 (88.2)
16	11 (11.8)
Upfront PTA to facilitate sheath insertion	10 (10.8)
Location of vascular complication	
Main access	87 (93.5)
Contralateral access	6 (6.5)
Complication management	
Covered balloon-expandable stent	93 (100)
Number of covered balloon-expandable stents	
One	78 (83.9)
Two or more	15 (16.1)
Type of covered balloon-expandable stent	
BeGraft	76 (81.7)
Atrium Advanta V12	17 (18.3)
Diameter of the stent, mm	9 [8; 9]
6 mm	1 (1.1%)
7 mm	7 (7.5%)
8 mm	35 (37.6%)
9 mm	32 (34.4%)
10 mm	16 (17.2%)
Unknown	2 (2.2%)
Length of the stent, mm	37 [37; 37]
27 mm	8 (8.6%)
28 mm	2 (2.2%)
30 mm	1 (1.1%)
37 mm	61 (65.6%)
38 mm	13 (14%)
39 mm	1 (1.1%)
57 mm	2 (2.2%)
59 mm	1 (1.1%)
Successful transcatheter heart valve delivery	91 (97.8)
Successful transcatheter heart valve implantation	91 (97.8)
Multiple valves	0 (0)
Aortic valve pre-dilation	58/91 (63.7)
Aortic valve post-dilation	37/91 (40.7)
Transcatheter heart valve implanted	91/93 (97.8)
Sapien XT	2/91 (2.2)
Sapien 3	19/91 (20.9)
Sapien 3 Ultra	37/91 (40.7)
Acurate neo	6/91 (6.6)
Acurate neo2	19/91 (20.9)
Evolut R	8/91 (8.8)
Size of implanted transcatheter heart valve	
23 mm	34/91 (37.4)
25 mm	12/91 (13.2)
26 mm	32/91 (35.2)
27 mm	3/91 (3.2)
29 mm	10/91 (11)

Data are shown as counts (%), mean ± SD (standard deviation) or median [25^th^–75 th percentiles]

*PTA* percutaneous transluminal angioplasty

Technical success, defined as successful delivery and implantation of the covered stent in the absence of residual bleeding, need for bailout surgery or intra-procedural death was achieved in 92 patients (98.9%). Successful THV delivery and implantation was achieved in 91 patients (97.8%). THV delivery was unsuccessful in 2 (2.2%) patients; in one patient, after combined upfront intravascular lithotripsy (IVL) and non-compliant (NC) balloon angioplasty of the ilio-femoral vessels and successful sheath placement, repeated advancement attempts of a balloon‐expandable THV led to partial extroversion of the valve frame, with ensuing vessel laceration. Despite placement of a stopping balloon in the abdominal aorta and implantation of several covered stents, refractory hemorrhagic shock leading to intraprocedural death occurred. In the second patient, despite successful sheath placement, THV valve advancement proved impossible and, following sheath removal, percutaneous placement of a covered stent was required to achieve hemostasis at the access site; the patient died on the 3rd postprocedural day, due to cardiogenic shock. Albeit very rare, these examples underscore the importance of achieving complete haemostasis in an effective and timely manner and illustrate the significant morbidity and mortality related to a failure in achieving these goals.

In-hospital clinical and echocardiographic findings are reported in Table 4. All-cause mortality was observed in four patients (4.3%). Besides the two aforementioned patients, one patient died due to septic shock (1.1%) and the other patient due to hemorrhagic shock (1.1%).

**Table 4:** In-hospital clinical and echocardiographic outcomes

Intra-procedural death	1 (1.1)
In hospital all-cause mortality	4 (4.3)
Cardiogenic shock	1 (1.1)
Hemorrhagic shock	2 (2.2)
Septic shock	1 (1.1)
Stroke	
Major	1 (1.1)
Minor	2 (2.2)
Periprocedural acute myocardial infarction	1 (1.1)
New permanent pacemaker	7 (7.5)
Acute kidney injury^a^	14 (15.1)
Red blood cell units transfused	0 [0; 2]
Intensive care unit length of stay, days	1 [1; 3]
Degree of paravalvular regurgitation	
Angiographic PVL ≥ 2	4 (4.3)
Echocardiographic PVL ≥ 2	2 (2.2)
Mean trans-prosthetic gradient, mmHg	11 [8; 15]
Maximal trans-prosthetic gradient, mmHg	20.6 [15.4; 28.7]
Post-procedural systolic pulmonary artery pressure, mmHg	39.9 ± 15.5
Minimal post-procedural hemoglobin value, gr/dL	8.4 [7.5; 10.2]

Data are shown as counts (%), mean ± SD (standard deviation) or median [25^th^–75 th percentiles]

*PVL* paravalvular leak

^a^Acute kidney injury was defined according to RIFLE criteria

### Access site and valve-related complications

The access-site and cardiac structural- and technical valve-related complications are shown in Table 5**.** Closure device failure was observed in 37 patients (39.8%), puncture-related bleeding in 21 patients (22.6%) and dissection or stenosis in 20 patients (21.5%). No conversion to surgery or valve embolization/migration was observed. One patient (1.1%) had an embolic coronary obstruction that was successfully managed through percutaneous transcatheter intervention.

**Table 5:** Access-site and cardiac structural- and technical valve-related complications

	*N* = 93
Procedural and technical valve-related complications	
Conversion to surgery	0 (0)
Valve embolization	0 (0)
Valve migration	0 (0)
Ectopic valve deployment	0 (0)
Cardiac structural complications	
Cardiac perforation	0 (0)
Cardiac tamponade	0 (0)
Coronary obstruction	1 (1.1)
Access-site related complications	
Closure device failure	37 (39.8)
Puncture related bleeding	21 (22.6)
Dissection/stenosis	20 (21.5)
Thrombosis	5 (5.4)
Perforation	7 (7.5)
Pseudoaneurysm	3 (3.2)

Data are shown as counts (%)

### Clinical and Doppler ultrasonographic follow-up

**Fig. 3:**
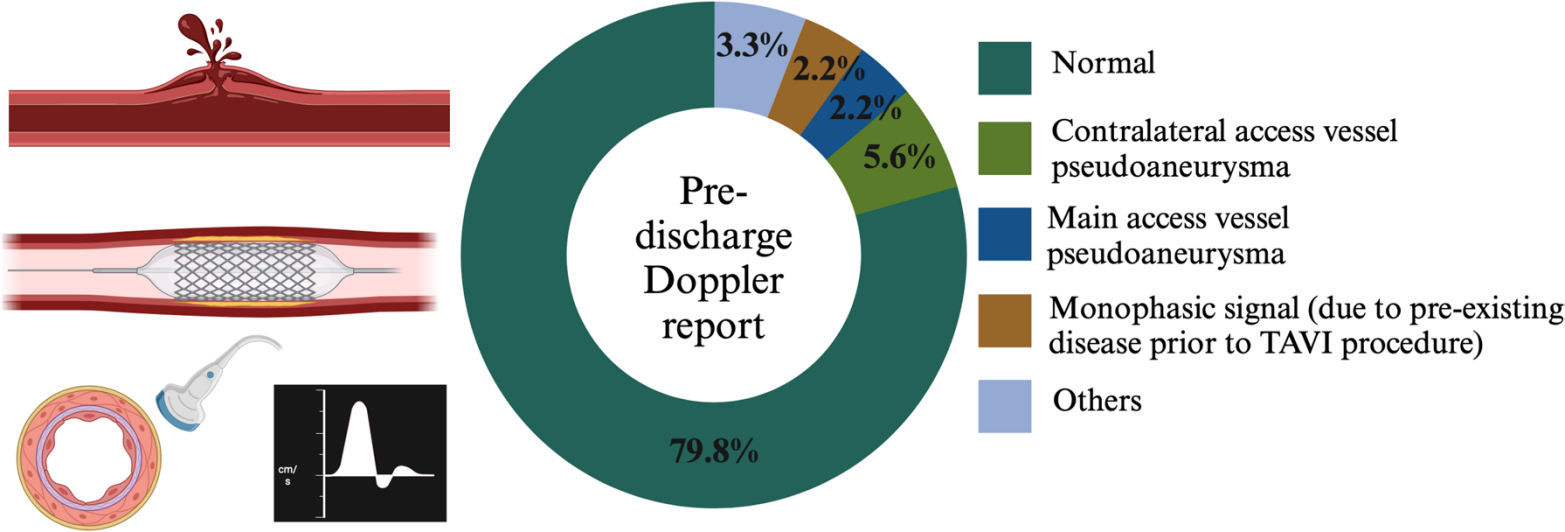
Findings of pre-discharge vascular Doppler ultrasonographic examinations in patients undergoing implantation of covered balloon-expandable stents for the treatment of access-site related vascular complications in the setting of transfemoral transcatheter aortic valve implantation

Median clinical follow-up was 455 [304; 798] days, with both in-hospital and 30-days mortality equaling 4.3%; importantly, no additional deaths and no repeat surgical or transcatheter peripheral vascular interventions were required during the critical time window comprised between patient discharge and 30-days follow-up. All-cause mortality equaled 15.1% at one year and 23.7% at the longest available clinical follow-up (Table 6). In-hospital and follow-up Doppler ultrasonographic data are shown in Table 6. In-hospital Doppler ultrasonographic examination was performed in 88/89 (98.9%) patients, with normal findings in 71 patients (79.8%), main access-related pseudoaneurysm in 2 patients (2.2%), contralateral access-related pseudoaneurysm in 5 patients (5.6%), main access related dissection in one patient (1.1%), main access arterio-venous fistula in one patient (1.1%), contralateral access arterio-venous fistula in one patient (1.1%) and a monophasic flow in two patients with advanced peripheral artery disease (2.2%) (Fig. 3). Clinical follow-up was available in 76/79 (96.2%) patients. Three patients were lost to follow-up. No cases of new-onset claudication, need for repeat surgical or transcatheter vascular interventions were recorded. Doppler ultrasonographic follow-up (92 [46; 101] days) was available in 43 (54.4%) patients and showed persistent stent patency in all patients with no signs of stent failure or additional pathological findings. Two patients with pre-existing advanced peripheral artery disease had a monophasic flow related to the underlying vascular disease but no signs of stent failure.

**Table 6:** Clinical and Doppler ultrasound follow-up

	*N* = 93
Follow-up, days	455 [304; 798]
In-hospital all-cause mortality	4 (4.3)
30-days all-cause mortality	4 (4.3)
Six-months all-cause mortality	11 (11.8)
One-year all-cause mortality	14 (15.1)
Overall all-cause mortality total	22 (23.7)
Pre-discharge Doppler ultrasound available	88/89 (98.9)
Pre-discharge Doppler ultrasound findings	
Normal	71 (79.8)
Main access vessel pseudoaneurysm	2 (2.2)
Contralateral access vessel pseudoaneurysm	5 (5.6)
Main access dissection	1 (1.1)
Main access arterio-venous fistula	1 (1.1)
Contralateral arterio-venous fistula	1 (1.1)
Monophasic signal	2 (2.2)
Clinical follow-up available	76/79 (96.2)
Lost to follow-up	3/89 (3.4)
Doppler ultrasound control follow-up, days	92 [46; 101]
Follow-up Doppler ultrasound available	43/79 (54.4)
Normal	41/43 (95.3)
Monophasic signal	2/43 (4.7)

Data are shown as counts (%), mean ± SD (standard deviation) or median [25^th^–75 th percentiles]

## Discussion

The present study sought to investigate immediate and mid- to long-term efficacy and safety of CBE stent implantation used for the management of access-related vascular complications in the setting of Tf-TAVI procedures. The major findings of the study can be summarized as follows: i) CBE stent implantation is highly effective in achieving complete hemostasis as well as vessel patency, with a technical success rate of 98.9%; ii) mid-term Doppler ultrasonographic and long-term clinical follow-up support the persistent efficacy and safety of CBE stent implantation in iliofemoral vessels, with no requirement for repeat transcatheter or surgical peripheral vascular interventions or clinically apparent stent failure during the available follow-up. Additionally, this is the first report confirming the efficacy and safety of the new generation single layer polytetrafluoroethylene (PTFE) BeGraft (Bentley InnoMed GmbH, Hechingen, Germany) CBE stent, used in the vast majority of procedures in the present registry, in the management of access-related vascular complications in the setting of Tf-TAVI procedures.

Besides continuous device iterations and increasing operator experience, which have already led to significant reductions in the rate of access-related major vascular complications, another promising option in preventing their occurrence is represented by ultrasound-guided puncture. By providing real-time images of important vessel anatomical features, such as location of the femoral artery bifurcation as well as presence and extension of atherosclerotic plaque and/or calcification, this technique facilitates the selection of an optimal puncture site. Although prospective, randomized studies supporting its use in the setting of Tf-TAVI are still lacking, there is growing evidence showing that use of this approach is associated with a significant reduction of vascular and bleeding complications associated with procedures requiring a femoral access [14].

Due to the lack of direct comparative studies, no clear recommendations are available regarding the optimal management strategy of vascular complications in the setting of TAVI procedures. Although surgical repair has represented in many centers the time-honored management strategy, due to the advanced age and comorbidities of patients undergoing TAVI procedures, it portends a non-negligible morbidity and mortality risk [15]. By allowing a rapid and effective vascular repair in the setting of large bore vascular access, endovascular approaches have become an attractive treatment strategy, which might even display potential advantages compared to conventional surgical repair [7].

Based on their release mechanism as well as engineering and design, covered stents can be classified into CSE and CBE. These two families display significant differences in terms of intrinsic physical properties, implantation characteristics as well as interaction with native vessel anatomy, which might translate into advantages and/or drawbacks in specific vascular territories. CBE stents display higher rigidity and radial stiffness and allow a more precise implantation and lesser foreshortening compared to CSE stents, which might be of particular interest in the proximity of large bifurcations. On the other hand, CSE stents are significantly more elastic and flexible, offer superior resistance to axial fatigue, which might translate into superior long-term mechanical stent integrity; the latter characteristic, might be particularly important in vascular territories subjected to cyclic flexion–extension movements, such as the common femoral artery. For this reason, the majority of previous reports investigating the use of covered stents in the setting of TAVI-related vascular complications have concentrated on CSE stents [9–12]. Taken together, these studies have reported high technical success rates [11] and excellent long-term patency rates of CSE stents [12]. On the other hand, comparative studies investigating efficacy and safety of balloon-expandable and self-expanding uncovered stents for the treatment of ilio-femoral lesions have shown somehow conflicting results [16, 17]. The Society for Cardiovascular Angiography and Interventions (SCAI) guidelines on device selection, give stronger recommendations to BE as compared to SE uncovered stents in aorto‐iliac arterial interventions [18], whereas the ESC guidelines give no specific recommendations regarding stent type [19]. The number of investigations evaluating clinical outcomes following CBE stent implantation is limited [20, 21], with the largest registry to date by Maurina et al. [20] retrospectively evaluating the outcomes of 78 patients undergoing implantation of the Atrium Advanta V12 (Getinge) CBE stents. The authors reported excellent technical success rates (96.2% as compared to 98.9% in the present registry) with bailout surgery required in only two patients; analogously to the present report, at a median follow-up of 429 (89–994) days [455 (304–798) days in the present registry], no cases of acute ipsilateral lower limb ischemia or need for repeat intervention were reported, thereby confirming the persistent excellent efficacy and safety of CBE stents in this setting (Supplementary Table 2).

Importantly, in the present registry, the vast majority (81.7%) of the patients underwent implantation of a BeGraft (Bentley) CBE stent; while there is a reasonable amount of data supporting the persistent efficacy and favorable long-term patency rate of the Advanta V12 (Getinge) CBE stent in the treatment of aorto-iliac occlusive disease [22], the present report is, to the best of our knowledge, the first to provide data regarding procedural efficacy and mid- to long-term clinical efficacy and safety following implantation of this novel CBE stent in ilio-femoral vessels in the setting of access-site-related vascular complications of Tf-TAVI procedures. The BeGraft (Bentley InnoMed GmbH, Hechingen, Germany) CBE stent is a Cobalt-Chrome (L-605), open-cell platform covered with a single-layer microporous ePTFE membrane (203 ± 25 μm) clamped at the proximal and distal stent ends, which has already shown encouraging results in terms of both clinical and angiographic efficacy as well as of safety profile in the management of perforations located in coronary vessels [23] as well as of iatrogenic access site related arterial side-branch injury [24]. The results of the present study extend these previous findings and confirm the excellent efficacy and safety of this novel stent platform for the treatment of access related vascular complications in the setting of TAVI procedures.

### Study limitations

The present report has a number of limitations. Despite its moderately large sample size, this is a single center, retrospective, observational study and a selection bias cannot be rule out. Moreover, although the reasonably long and thoroughly conducted clinical follow-up is reassuring regarding the absence of hard clinical endpoints, only 54.4% of the patient collective underwent a Doppler ultrasonographic follow-up and the occurrence of asymptomatic stent failure (stent fracture or restenosis) might be underestimated.

## Conclusions

CBE stent implantation for the treatment of access site-related vascular complications after Tf-TAVI is associated with excellent technical success rates, optimal short- to mid-term patency rates as well as satisfactory long-term clinical outcomes. Due to the combination of optimal acute as well as mid- to long-term efficacy and safety, the results of the present study, the largest to evaluate patient clinical outcomes following the use of CBE stents to date, support the use of CBE stents for the management of access-related vascular complications in the setting of TAVI procedures.

## Supplementary Information

Below is the link to the electronic supplementary material.

Supplementary file1 (DOCX 19 KB)

## Abbreviations

CBE: Covered balloon-expandable
CSE: Covered self-expanding
CT: Computed tomography
IVL: Intravascular lithotripsy
PTA: Percutaneous transluminal angioplasty
TAVI: Transcatheter aortic valve implantation
Tf: Transfemoral
THV: Transcatheter heart valve
VARC-3: Valve Academic Research Consortium-3
