# Supplementary material for: Mid- to long-term outcomes of covered balloon-expandable stent implantation for the management of vascular injuries in patients undergoing transfemoral transcatheter aortic valve implantation: the BE-SAFE Registry: for the RECOVER (REsults after percutaneous interventions with COVERed stents) Investigators
Source: Clin Res Cardiol. 2025 May 15;115(7):1120–30. doi: 10.1007/s00392-025-02651-2 (PMC13249666; doi:10.1007/s00392-025-02651-2)
Supplement: Supplementary file 1 — Supplementary file1 (DOCX 19 KB) [file 392_2025_2651_MOESM1_ESM.docx]

**Supplementary table 1. Computed tomography analysis of the aortic valve.**

| Aortic valve measurements n = 83/93 * | |
| --- | --- |
| Aortic valve minimal diameter, mm | 20.8±2.5 |
| Aortic valve maximal diameter, mm | 26.9±2.8 |
| Aortic valve mean diameter, mm | 23.9±2.4 |
| Aortic valve area, mm^2^ | 428 [373; 486] |
| Aortic valve perimeter, mm | 76±7.8 |
| Left main height, mm | 13.1±2.5 |
| Right coronary artery height, mm | 17 [15; 18] |
| Aortic valve calcification severity | 83/93 (89.2) |
| - Mild | 27/83 (32.5) |
| - Moderate | 37/83 (43.4) |
| - Severe | 20/83 (24.1) |

* Reported only for native valve anatomy; ViV procedure was performed in the remaining 10 patients

mm = millimeters; mm^2^ = square millimeters

| **Supplementary Table 2. Available studies investigating percutaneous stent implantation for the management of access site vascular complication following transcatheter aortic valve implantation** | | | | |
| --- | --- | --- | --- | --- |
| Author | Year | Stent Type | No. Patients | Success |
| ^10^Perrin et al | 2015 | 10 patients - Self-expanding uncovered nitinol stents (S.M.A.R.T.® [Cordis Corporation, Hialeah, FL USA] and Misago® [Terumo Corporation, Tokyo, Japan]).  2 patients - Self-expanding elgiloy stents covered with a layer of polyethylene terephthalate (WALLGRAFT®; Boston Scientific, Natick, MA, USA) or self-expanding nitinol stents covered with polytetrafluoroethylene (Fluency®; Bard Peripheral Vascular, Phoenix, AZ, USA) | **13** | 85% |
| ^11^Sedaghat et al. | 2016 | Self-expanding nitinol stent graft (polytetrafluoroethylene) | **96** | 96.9% |
| ^12^Sedaghat et al. | 2019 | 31 patients - Self-expanding Viabahn stent graft (Gore Inc., Flagstaff, AZ, USA)  40 patients - Fluency stent graft (C.R. BARD Inc., Murray Hill, NJ, USA) | **71** | 100% |
| ^20^ Drouelle et al. | 2019 | 21 Patients - Balloon-expandable covered stents (Atrium Advanta V12; Getinge)  3 Patients - Balloon Expandable Vascular Covered Stent (Lifestream™)  1 Patient - Self-expandable covered stent (Fluency™ Plus Endovascular Stent Graft) | **26** | 92.3% |
| ^9^ Ben Abdallah et al. | 2020 | Covered self-expandable nitinol stents | **59** | 97% |
| ^19^ Maurina et al. | 2022 | Balloon-expandable covered stents (Atrium Advanta V12; Getinge) | **78** | 96.2% |
| ^7^Meertens et al. | 2023 | Epic (Boston-Scientific, Marlborough, Massachusetts)  or  Gore-Viabahn (W. L. Gore & Associates) | **15** | 93.3% |
| Alvarez-Covarrubias et al. | 2025 | 76 patients - Balloon-expandable single layer polytetrafluoroethylene covered stents (BeGraft, Bentley InnoMed GmbH, Hechingen,Germany)  17 patients - Balloon-expandable covered stents (Atrium Advanta V12; Getinge) | **93** | 98.9% |
